# SAMBA HIV Semiquantitative Test, a New Point-of-Care Viral-Load-Monitoring Assay for Resource-Limited Settings

**DOI:** 10.1128/JCM.00593-14

**Published:** 2014-09

**Authors:** Allyson V. Ritchie, Ines Ushiro-Lumb, Daniel Edemaga, Hrishikesh A. Joshi, Annemiek De Ruiter, Elisabeth Szumilin, Isabelle Jendrulek, Megan McGuire, Neha Goel, Pia I. Sharma, Jean-Pierre Allain, Helen H. Lee

**Affiliations:** a Diagnostic Development Unit, Department of Haematology, University of Cambridge, Cambridge, United Kingdom; b Barts and The London NHS Trust, London, United Kingdom; c Médecins Sans Frontières, Paris, France; d Department of Genitourinary Medicine and HIV, Guy's and St Thomas' NHS Foundation Trust, London, United Kingdom; e Division of Transfusion Medicine, Department of Haematology, University of Cambridge, Cambridge, United Kingdom

## Abstract

Routine viral-load (VL) testing of HIV-infected individuals on antiretroviral therapy (ART) is used to monitor treatment efficacy. However, due to logistical challenges, implementation of VL has been difficult in resource-limited settings. The aim of this study was to evaluate the performance of the SAMBA semi-Q (simple amplification-based assay semiquantitative test for HIV-1) in London, Malawi, and Uganda. The SAMBA semi-Q can distinguish between patients with VLs above and below 1,000 copies/ml. The SAMBA semi-Q was validated with diluted clinical samples and blinded plasma samples collected from HIV-1-positive individuals. SAMBA semi-Q results were compared with results from the Roche COBAS AmpliPrep/COBAS TaqMan HIV-1 test, v2.0. Testing of 96 2- to 10-fold dilutions of four samples containing HIV-1 subtype C as well as 488 samples from patients in the United Kingdom, Malawi, and Uganda yielded an overall accuracy for the SAMBA semi-Q of 99% (95% confidence interval [CI], 93.8 to 99.9%) and 96.9% (95% CI 94.9 to 98.3%), respectively, compared to to the Roche test. Analysis of VL data from patients in Malawi and Uganda showed that the SAMBA cutoff of 1,000 copies/ml appropriately distinguished treated from untreated individuals. Furthermore, analysis of the viral loads of 232 patients on ART in Malawi and Uganda revealed similar patterns for virological control, defined as either <1,000 copies/ml (SAMBA cutoff) or <5,000 copies/ml (WHO 2010 criterion; WHO, *Antiretroviral Therapy for HIV Infection in Adults and Adolescents: Recommendations for a Public Health Approach*, 2010). This study suggests that the SAMBA semi-Q has adequate concurrency with the gold standard measurements for viral load. This test can allow VL monitoring of patients on ART at the point of care in resource-limited settings.

## INTRODUCTION

There have been steady improvements in scaling up access to antiretroviral therapy (ART) in resource-limited countries (1). There appear to be fewer new infections, and AIDS-related deaths have decreased over the past decade. While these achievements are remarkable, there remains a large unmet need, given that 34 million people are living with HIV/AIDS globally, most of whom live in sub-Saharan Africa.

Effective ART not only improves the survival of individuals infected with HIV but also prevents transmission (2). The global public health community is therefore committed to achieving universal access to HIV treatment, with a target of increasing the availability of ART to 15 million people by the end of 2015 (3). Effective ART suppresses HIV replication, which is measured by monitoring plasma viral load (VL), specifically looking at potential adherence or treatment failure. VL monitoring prolongs the duration of first-line regimens by preventing unnecessary switches to more complex and expensive second-line regimens that result in increased costs and a decrease in drug options (4, 5), and it can reduce the delay in switching to second-line drugs or patient counseling, resulting in better patient outcomes (6, 7). Studies have shown that sites with VL monitoring have lower mortality rates (6) and better justification of switching to second-line drug regimens than sites using only CD4 monitoring (4).

Quantitative VL monitoring is the standard for assessing care of patients receiving ART in resource-rich settings (8, 9). In resource-poor settings, however, laboratory diagnostics are often available only at centralized laboratories in major cities due to the complexity of the technology and to the infrastructure and trained personnel required for the tests (10–12). As ART programs have scaled up, there has been a significant effort to decentralize care to local primary health centers, which have basic services and limited infrastructure. To access VL testing, peripheral HIV treatment facilities must transport patient specimens to central laboratories under optimal conditions within a limited period of time, followed by testing and return of results. This results in increased costs of service delivery and unacceptable delays in obtaining test results, with consequent losses to follow-up (13). A priority focus area of the Treatment 2.0 initiative is therefore the development of affordable, reliable, quality-assured, point-of-care molecular diagnostic platforms (14).

The SAMBA semi-Q (simple amplification-based assay semiquantitative test for HIV-1) has been developed as a robust, simple, and relatively rapid point-of-care test to distinguish between patients with VLs above and below 1,000 copies/ml within 90 min. The main advantages of the SAMBA include visual detection of the result and robust reagents, which are stable at high temperatures and humidity. All reagents required are preloaded in single-use, disposable cartridges to ensure that the system is easy to use and to prevent potential contamination of the laboratory with amplified product. The chemistry is based on the SAMBA qualitative test and can therefore detect all known HIV-1 subtypes (15). In consultation with experts in the field of ART provision in low- and middle-income countries (LMIC), Médecins Sans Frontières (MSF) concluded that a viral load of 1,000 copies/ml was the level most frequently used as the trigger for clinical intervention. Furthermore, the threshold for detecting treatment failure was lowered in South Africa in April 2010 from 5,000 to 1,000 copies/ml, and this has been implemented at various South African sites (5, 16). WHO also updated their guidelines in June 2013 and now define treatment failure as a persistently detectable viral load exceeding 1,000 copies/ml (8) rather than 5,000 copies/ml (17). Ideally, patients on ART for more than 6 months with a VL of >1,000 copies/ml should be counseled for adherence and retested 3 months later as per the WHO guidelines. If the VL remains >1,000 copies/ml at follow-up, this may indicate treatment failure. Therefore, it is important that patients with VLs of ≥1,000 copies/ml be identified by SAMBA semi-Q. On the other hand, it is also important that individuals with low VLs not be identified, as such results may be due to “blips.” VL blips are defined as intermittent episodes of detectable low VL (50 to 1,000 copies), which return to undetectable levels without any intervention (18, 19). This study evaluated the accuracy and performance of the SAMBA semi-Q with a cutoff of 1,000 copies/ml compared to gold standard viral load testing.

## MATERIALS AND METHODS

### Determination of VL with SAMBA semi-Q.

**FIG 1:**
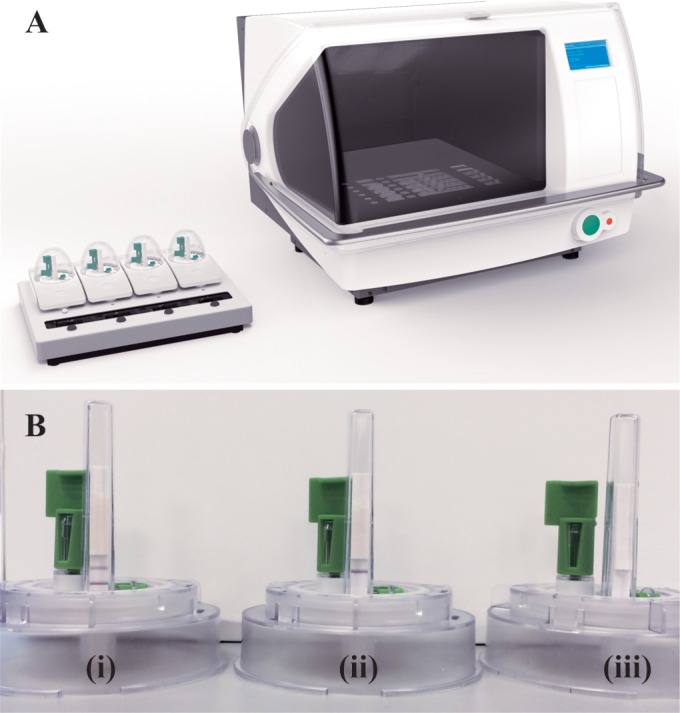
SAMBA semi-Q system. (A) SAMBAprep (right) and SAMBAamp (left) instruments. (B) SAMBAamp cartridge showing results for (i) >1,000 and (ii) <1,000 copies/ml and (iii) invalid results.

RNA was extracted from 200 μl of plasma using the SAMBA sample preparation instrument, SAMBAprep (Fig. 1A). The result was read visually after isothermal amplification and dipstick detection within the SAMBAamp instrument (Fig. 1A). The presence of a control line indicates a valid test, and the test line on the dipstick indicates a VL of >1,000 copies/ml, whereas the absence of the test line indicates a VL of <1,000 copies/ml. The absence of both lines indicates an invalid test (Fig. 1B). This assay does not detect HIV-2 and should not be used for diagnosis of HIV-1 infection.

### Preparation of dilution panels of HIV-1 subtype C samples for validation of the SAMBA semi-Q cutoff of 1,000 copies/ml.

We obtained four surplus samples from blood donors identified as positive for antibodies to HIV-1 from the National Blood Transfusion Centre in Windhoek, Namibia. Viral genotyping and VL quantification of the samples with the Roche COBAS AmpliPrep/COBAS TaqMan HIV-1 test, v2·0 (Roche TaqMan v2), were performed at The Royal London Hospital. Serial 2- to 10-fold dilutions of the four samples were prepared in HIV-negative plasma to achieve HIV-1 RNA concentrations ranging from 3 (0.48 log_10_) to 222,238 (5.35 log_10_) copies/ml according to the quantification by Roche TaqMan v2. Four replicates of each dilution were tested with the SAMBA semi-Q by two operators in-house to assess the precision and accuracy of the test. A total of 96 dilutions were tested.

### Data analysis.

The limit of accuracy for Roche TaqMan v2 is ±0.3 log_10_ relative to the VL readout obtained, according to the package insert. For the purpose of the present study, we therefore considered that any VL quantification by Roche TaqMan v2 within 0.3 log_10_ of the SAMBA semi-Q cutoff of 1,000 (3 log_10_) copies/ml, corresponding to a range of 500 to 2,000 copies/ml, was concordant with the SAMBA semi-Q result.

### In-house blinded testing of clinical samples.

Plasma samples from 134 HIV-1-infected individuals attending The Royal London Hospital (34 patients) or St Thomas' Hospital (100 patients) in London were rendered anonymous and provided blinded. The plasma samples were stored at −80°C until tested in-house with the SAMBA semi-Q. Roche TaqMan v2 results and HIV-1 subtype information (if available) were provided by both hospitals after the SAMBA semi-Q testing was completed. In addition, two external quality assessment (EQA) subtype panels from Rush University, RNA004XPA and RNA004XPB, containing 84 samples per panel (including subtypes A, A/E, A/G, C, D, F, and G) at 2,500 and 25,000 copies/ml, were tested blinded in-house.

### Field testing of SAMBA semi-Q in Malawi and Uganda.

Field testing of the SAMBA semi-Q was performed at ART provision program centers run by MSF in Malawi (Chiradzulu District Hospital) and Uganda (Arua Regional Referral Hospital). The Chiradzulu HIV program, which was established in 2000 and currently monitors 25,000 patients on ART, has been described in detail previously (20, 21). The HIV program in Arua was established in 2002 and follows 7,000 HIV-infected individuals on ART (22).

Samples were collected both from consecutive patients attending the HIV clinics, including those on ART, and from pre-ART patients in order to obtain a wide range of VLs. Plasma was separated from whole-blood specimens (10 ml) within 4 h of collection and tested fresh. In Malawi, the first 117 samples were sent to Cambridge for testing while the local technician was trained. The remaining 83 samples were tested on site by a trained MSF technician. In Uganda, the first 120 samples collected were tested on site by the MSF technician, and the remaining 34 samples were shipped to Cambridge for testing due to time constraints. All 354 samples from Malawi and Uganda were tested with Roche TaqMan v2 at The Royal London Hospital. In the case of a discrepancy between Roche TaqMan v2 and SAMBA semi-Q, the remaining frozen plasma was tested using the Abbott RealTime HIV-1 assay (Abbott RealTime) by an independent laboratory.

### Analysis of VL distribution among patients in Malawi and Uganda according to available clinical information.

Patient records were accessed, after consent was obtained, for the 354 patients in Malawi and Uganda and were analyzed after all testing was complete. The VLs of the 284 treated patients were compared with those of the 70 treatment-naive individuals in order to determine the VL spread. In addition, the VLs of 232 patients on ART for 0.5 to 9 years were stratified according to treatment duration and used to compare the number of individuals defined as virologically suppressed according either to the 2010 WHO guidelines (17) or to the SAMBA semi-Q cutoff.

### Research ethics.

The study was performed in accordance with the Declaration of Helsinki. Ethical approval was obtained from the National Health Sciences Research Committee, Ministry of Health and Population, for Chiradzulu Hospital (Malawi), from the Uganda National Council for Science and Technology for Arua District Hospital (Uganda), and from the Research Ethics Committee, NRES-London, for St Thomas' Hospital (United Kingdom). Nucleic acid testing of blood donor samples is approved in Namibia, and the four donors in the present study were informed of potential additional testing. Surplus samples obtained from patients known to be infected with HIV-1 and submitted to The Royal London Hospital for routine monitoring were retrieved before being discarded, rendered anonymous, and provided blinded for the purpose of test validation; the use of samples in this manner, strictly for the purpose of diagnostic assay validation, does not fall under the requirements of research ethics for the organizations in which they originated.

## RESULTS

### Validation of the accuracy of the SAMBA semi-Q cutoff.

All 52 dilutions of the four Namibian samples (all HIV-1 subtype C) containing >1,000 (>3.0 log_10_) copies/ml according to Roche TaqMan v2 were correctly identified as such with SAMBA semi-Q, and 43 of 44 (98%) dilutions containing <1,000 copies/ml according to Roche TaqMan v2 were similarly correctly identified by SAMBA semi-Q (Table 1). One of the dilutions that tested negative by SAMBA semi-Q but according to Roche TaqMan v2 should contain 1,211 (3.08 log_10_) copies/ml and two of the dilutions found to be positive by SAMBA semi-Q that should contain 606 (2.78 log_10_) copies/ml according to Roche TaqMan v2 were considered concordant because of the accuracy limits of the TaqMan assay (see Materials and Methods). Therefore, the overall concordance between SAMBA semi-Q and Roche TaqMan v2 for these 96 sample dilutions was 99% (95/96; 95% confidence interval [CI], 93.8 to 99.9).

**TABLE 1:** Validation of the SAMBA semi-Q cut-off with diluted plasma samples containing HIV-1 subtype C^*a*^

Sample	No. of HIV-1 RNA copies/ml	% with positive SAMBA HIV-1 semi-Q result (>1,000 copies/ml)
1	151,114	100
	15,111	100
	1,511	100
	756	0
	151	0
	15	0
2	30,543	100
	3,054	100
	1,527	100
	305	0
	31	0
	3	0
3	222,238	100
	22,224	100
	2,222	100
	1,111	100
	222	25
	22	0
4	121,102	100
	12,110	100
	1,211	75
	606	50
	121	0
	12	0

a Four plasma samples were serially diluted to achieve concentrations of viral RNA ranging from 3 (0.48 log_10_) to 222,238 (5.35 log_10_) copies/ml according to quantification with Roche TaqMan v2. Four replicates of each dilution were tested with the SAMBA semi-Q.

### Specificity.

The specificity of the SAMBA HIV-1 assay was evaluated by testing 216 plasma specimens from HIV-1-seronegative patients. The assay was not reactive for all 216 specimens, and the SAMBA HIV-1 assay specificity was calculated to be 100% (216/216) (95% CI, 98.6 to 100%). The specificity of the SAMBA semi-Q was further evaluated using a panel of 43 specimens obtained from samples containing the following viruses, microorganisms, or antibodies from autoimmune disorders: hepatitis A virus (2 samples), hepatitis B virus (24 samples), hepatitis C virus (3 samples), cytomegalovirus (CMV) (2 samples), and HIV-2, human T-cell leukemia virus type 1 (HTLV-1), HTLV-2, the causative agent of syphilis, antinuclear antibodies (ANA), Chlamydia trachomatis, Neisseria gonorrhoeae, Propionibacterium acnes, Staphylococcus aureus, Candida albicans, Staphylococcus epidermis, and Streptococcus pyogenes (1 sample of each). All tested negative by the SAMBA semi-Q.

### Potentially interfering substances.

The susceptibility of the SAMBA semi-Q to interference by elevated levels of endogenous substances and drugs commonly prescribed to HIV-1-infected individuals was evaluated. HIV-1-negative samples and samples containing 1,000 and 2,000 IU/ml of HIV-1 RNA were tested in the presence of the following substances: hemoglobin (500 mg/dl), triglycerides (3,000 mg/dl), bilirubin (20 mg/dl), and human DNA (0.4 mg/dl). No interference in the performance of the SAMBA semi-Q was observed. Testing of ART drugs at concentrations in excess of 1.5 times the peak plasma level (*C*_max_) was performed using the following: abacavir/lamivudine, efavirenz/tenofovir/emtricitabine, lopinavir/ritonavir, lamivudine/zidovudine, nevirapine, ribavirin, and saquinavir. No interference in the performance of the SAMBA semi-Q was observed.

### In-house blinded testing of clinical samples.

A total of 134 HIV-1-positive plasma samples from 100 male and 34 female patients attending two London hospitals were tested with the SAMBA semi-Q in a blinded manner in-house. The SAMBA semi-Q was concordant with Roche TaqMan v2 for 131 of the 134 samples when four specimens (VLs of 800, 948, 1,285, and 1,507 copies/ml) were considered concordant because of the accuracy limits of the TaqMan assay (Table 2). One of 35 samples found to contain >2,000 (3.3 log_10_) copies/ml by Roche TaqMan v2 was negative by SAMBA semi-Q (2,508 copies/ml), and 2 of 95 samples found to contain <500 (2.7 log_10_) copies/ml by the Roche TaqMan assay tested positive by SAMBA semi-Q (53 and 252 copies/ml). The concordance between SAMBA semi-Q and Roche TaqMan v2 was thus 97.8% (131/134; 95% CI, 93.3 to 99.5). Viral subtype data provided for 76 of these samples after the SAMBA semi-Q testing was completed revealed the following subtype distribution: 44.7% B, 18.4% C, 10.5% CRF02_AG, 5.3% A, 2.6% CRF01_AE, F, G, G/CRF02_AG, CRF11_cpx/CRF13_cpx, and A/AE, and 1.3% D, CRF06_cpx, D/A, and D/F. The subtypes for only two of the three discrepant samples (B and A/D) were available. In addition, a blinded EQA subtype panel provided by Rush University was tested, and all 168 samples (84 at 2,500 and 84 at 25,000 copies/ml), including subtypes A, CRF01_AE, CRF02_AG, C, D, F, and G, were successfully detected by the SAMBA semi-Q.

**TABLE 2:** Comparison of the SAMBA semi-Q and Roche TaqMan v2 results for 488 clinical samples from London (*n* = 134), Malawi (*n* = 200), and Uganda (*n* = 154)^*a*^

Sample source and SAMBA Semi-Q result (copies/ml)	No. with result with Roche TaqMan v2 (copies/ml)	
	<1,000	>1,000
London		
<1,000	95	1
>1,000	2	36
Malawi		
<1,000	146	0
>1,000	4	50
Uganda		
<1,000	91	5
>1,000	3	55

a Concordance between the two tests was 96.9% (473/488) overall, 97.8% (131/134) in London, 98.0% (196/200) in Malawi, and 94.8% (146/154) in Uganda.

### Field testing of SAMBA semi-Q in Malawi and Uganda.

A total of 200 samples collected in Chiradzulu, Malawi, were from 72 men and 128 women, with the patients ranging in age from 18 to 61 years. Four patients were assigned an ID but withdrew from the study with no sample being collected. The 154 samples collected in Arua, Uganda, were from 68 men and 86 women, with ages ranging from 18 to 71 years. Overall, 70 patients (19.8%) were ART naïve, and 284 (80.2%) had been on ART for a period of 1 month to 10 years at the time of testing.

A total of 196 of the 200 samples collected in Malawi were correctly classified by SAMBA semi-Q (Table 2), with five samples (VLs of 601, 651, 922, 1,539, and 1,599 copies/ml) being included as concordant because of the accuracy limits of Roche TaqMan v2. For Uganda, 146 of the 154 samples were concordant between SAMBA semi-Q and Roche TaqMan v2, with one sample (VL of 1,061 copies/ml) being classified as such as a result of the accuracy limits of the TaqMan assay. The concordance between SAMBA semi-Q and Roche TaqMan v2 was thus 96.6% overall, 98.0% in Malawi, and 94.8% in Uganda (Table 2).

**FIG 2:**
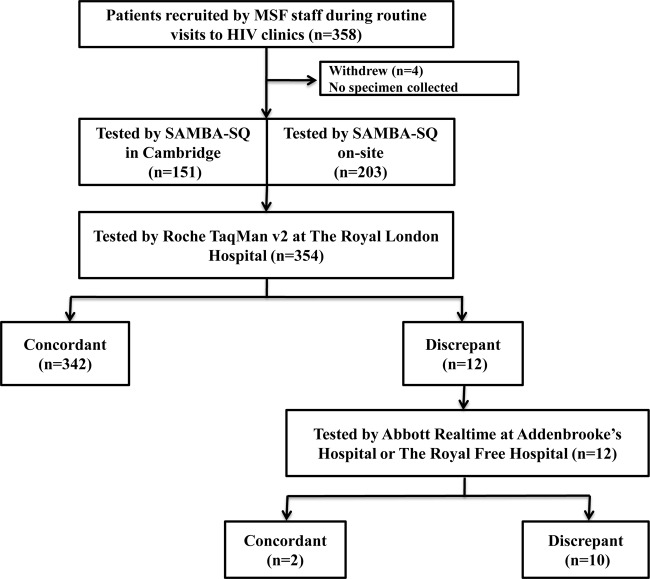
Field testing algorithm for the SAMBA semi-Q with 354 samples collected in Malawi and Uganda and summary of results. All samples were tested with the SAMBA semi-Q and Roche TaqMan v2. Twelve samples were discrepant between SAMBA and Roche and were tested with Abbott RealTime. Ten of the 12 samples were discrepant between SAMBA and Abbott, and two were discrepant between Abbott and Roche.

Taking into consideration all data, 18 samples (5%) were discrepant between SAMBA and Roche, including six samples within the ±0.3 log_10_ accuracy of the SAMBA semi-Q cutoff. Twelve of the 354 samples (3.4%) were truly discordant, with a VL outside the ±0.3 log_10_ accuracy of the SAMBA semi-Q cutoff. These 12 samples (four from Malawi and eight from Uganda) included seven found to contain <500 (2.7 log_10_) copies/ml and five found to contain >2,000 (3.3 log_10_) copies/ml by Roche TaqMan v2 (Fig. 2 and Table 2). These 12 discordant samples were retested with Abbott RealTime at one of two independent laboratories and in a blinded manner with regard to the SAMBA semi-Q and Roche TaqMan v2 results. The Abbott RealTime results were concordant with the Roche TaqMan v2 results for 10 of the 12 samples (Fig. 2). Two of the original five discrepant samples found to contain >2,000 (3.3 log_10_) copies/ml by Roche TaqMan v2 were found to contain <1,000 copies/ml by Abbott RealTime.

### Analysis of VL distribution among African patients according to available clinical information.

**FIG 3:**
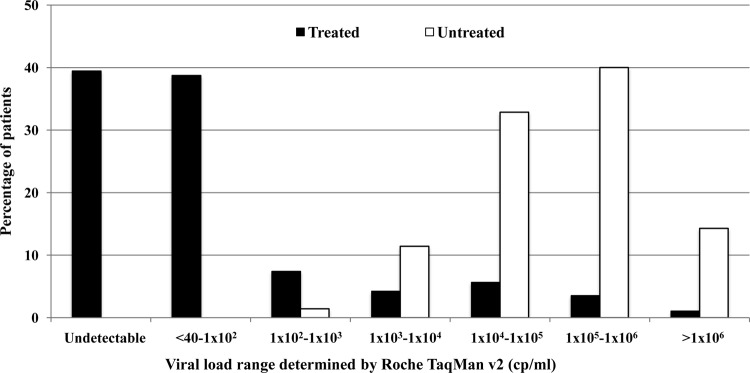
Distribution of VL among 284 patients receiving ART and 70 untreated individuals in Malawi and Uganda. VL was determined with Roche TaqMan v2.

The VLs of the 284 treated patients from Malawi and Uganda ranged from 0 to 2.6 × 10^6^ copies/ml, whereas those of the 70 treatment-naive individuals ranged from 1.2 × 10^2^ to >1.0 × 10^7^ copies/ml (Fig. 3). In both Malawi and Uganda, the SAMBA semi-Q cutoff value of 1,000 copies/ml clearly separated individuals in the untreated group from those in the treated group.

**FIG 4:**
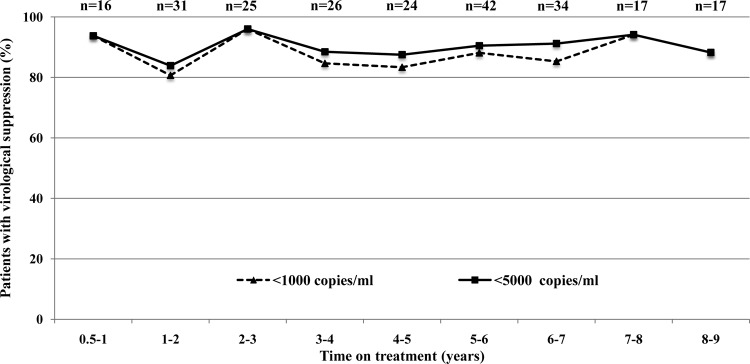
Virological suppression according to the SAMBA semi-Q cutoff and 2013 WHO guidelines (1,000 copies/ml [8]) or 2010 WHO guidelines (5,000 copies/ml [17]) in 232 patients on ART for various numbers of years in Uganda and Malawi.

The VL results obtained with Roche TaqMan v2 for 232 patients on ART for 0.5 to 9 years were stratified according to duration of therapy, and virological suppression was defined as either <1,000 copies/ml (SAMBA semi-Q cutoff) or <5,000 copies/ml (2010 WHO guidelines [17]) (Fig. 4). With either definition, 93.8% of individuals manifested virological suppression after 0.5 to 1 year on treatment. The percentage of individuals with suppression fluctuated between 80.6 and 96.0% (with a definition of 1,000 copies/ml) or between 83.9 and 96.0% (with a definition of 5,000 copies/ml) for treatment durations of 1 to 9 years. Similar results were obtained with the two definitions of virological suppression, because only six individuals (2.6%) on treatment for 0.5 to 9 years had VLs between these two values.

## DISCUSSION

The SAMBA semi-Q cutoff was validated for accuracy in comparison with the Roche TaqMan v2 test with the use both of diluted clinical samples and of blinded plasma samples in house and in the field in two public-sector ART provision programs in Africa. This study demonstrated that the SAMBA semi-Q is able to effectively differentiate between patients with VLs above and below the defined threshold of 1,000 copies/ml. The current gold-standard VL assays have given accuracy acceptance criteria of ±0.3 log_10_ for Roche TaqMan v2 and ±0.25 log_10_ for Abbott RealTime relative to the nominal input concentration (23). For the present evaluations, we selected Roche TaqMan v2 as the gold standard, as it is widely used. Any samples found to contain 500 to 2,000 (2.7 log_10_ to 3.3 log_10_) copies/ml by this assay were therefore considered concordant with the SAMBA semi-Q result, given that the true VL might lie on either side of the cutoff. A dilution series for four samples containing HIV-1 subtype C tested by two operators revealed excellent concordance (99.0%). The subtype coverage of SAMBA was evaluated using a blinded EQA panel from Rush University, in which the SAMBA semi-Q detected 100% of the samples, including subtypes A, CRF01_AE, CRF02_AG, C, D, F, and G. The SAMBA semi-Q cutoff was further validated in house with a blinded panel of clinical samples, including HIV-1 subtypes A to G and a range of recombinants, yielding an accuracy of 97.8% and showing that the accuracy extends over a wide variety of viral subtypes. The reproducibility and accuracy of the SAMBA semi-Q with fresh clinical samples collected in the field were evaluated by a trained field technician in each of two public-sector ART provision programs in Malawi and Uganda. The data were again compared with Roche TaqMan v2 results, revealing an overall concordance of 96.6%. In total, the concordance of SAMBA semi-Q with Roche TaqMan v2 as determined with clinical samples from London and Africa was 97.3% (568/584; 95% confidence interval, 95.6 to 98.3), indicating that the performance of the SAMBA semi-Q is in line with that of the available commercial assays. Importantly, the SAMBA semi-Q was performed on site in Malawi and Uganda by trained MSF technicians, showing that it is simple enough to serve as an appropriate diagnostic platform for use in district hospitals in sub-Saharan Africa.

Our data suggest that SAMBA semi-Q, with its cutoff of 1,000 (3 log_10_) copies/ml, is likely to prove a useful tool for assessment of the efficacy of ART and for identifying patients either who have developed virological failure and possible antiretroviral resistance or who have been infected with a drug-resistant strain of HIV-1. This cutoff level should also help to minimize unnecessary treatment switching due to viral blips. In addition, given that the test can be performed in the field within 90 min, the patient can remain on site and appropriate action can be taken during the same visit. This is hugely beneficial, given the fact that patients frequently face very long journeys to and from health centers. In Khayelitsha, South Africa, the ability of ART to reduce VL to an undetectable level was found to correlate with the timing of viral detection and the subsequent treatment adherence support provided (4). The availability of an easy-to-use, semiquantitative, and inexpensive rapid test to detect virological failure would therefore be expected to make an important contribution to optimization of first- and second-line treatment in resource-constrained countries (7).

Our analysis of VL distribution in African patients indicated that the SAMBA semi-Q cutoff is able to reliably differentiate patients on effective ART from nontreated patients as well as identifying patients with virological failure according to current WHO guidelines (8). Analysis of the VL of 232 patients on ART for 0.5 to 9 years showed that the temporal pattern for virological failure as defined in the SAMBA semi-Q model and current WHO guidelines (>1,000 copies/ml) was highly similar to that observed with the 2010 WHO guidelines (>5,000 copies/ml [17]).

One key advantage of the SAMBA system is that it relies on visual detection of nucleic acid on a test strip, with a readout similar to that of an HIV antibody rapid test. The processed test strip can be shown to the patient as a reinforcement tool. However, although the difference in signal strengths between positive and negative results for SAMBA semi-Q is greater than that seen with many rapid tests, there remains the possibility of transcription errors or misinterpretation of results by operators in the field. This limitation will be overcome by the development of SAMBA 2, a fully integrated system in the form of a small bench-top instrument, where the sample is introduced and the result appears on a screen or is printed on paper.

The SAMBA semi-Q is a semiquantitative test for differentiation between patients with a VL above or below 1,000 copies/ml, which may be regarded as a limitation of the assay, given that currently available commercial tests provide a numerical readout. Although these readouts appear to be accurate, the accuracy of the results differs between tests, being ±0.3 log_10_ for Roche TaqMan v2 and ±0.25 log_10_ for Abbott RealTime. Furthermore, the Abbott assay consistently reads lower than the Roche test (23), which was apparent in our analysis of discrepant samples. These VL numbers can be useful for tracking the initial virological response of individuals to treatment and are routinely reported to patients in industrialized countries. However, in LMICs, where mass treatment monitoring is required but is currently not available, the main need of the clinician is to identify individuals who are not responding to treatment and act accordingly as soon as possible. MSF have implemented the SAMBA semi-Q for routine use at one site in Arua and two sites in Chiradzulu since August 2013. Patients are monitored twice per year, and results are given at the same visit. Those with a VL of >1,000 copies/ml are counseled for adherence reinforcement, and if they still have a viral load of >1,000 copies/ml at the next visit, they are switched to second-line therapy.
